# Targeting G-protein coupled receptor-related signaling pathway in a murine xenograft model of appendiceal pseudomyxoma peritonei

**DOI:** 10.18632/oncotarget.22455

**Published:** 2017-11-06

**Authors:** Ashok K. Dilly, Brendon D. Honick, Yong J. Lee, Zong S. Guo, Herbert J. Zeh, David L. Bartlett, Haroon A. Choudry

**Affiliations:** ^1^ Department of Surgery, University of Pittsburgh Medical Center, Pittsburgh PA 15232, USA; ^2^ Department of Pharmacology and Chemical Biology, University of Pittsburgh Medical Center, Pittsburgh PA 15232, USA

**Keywords:** MUC2, xenograft, pseudomyxoma peritonei, COX-2, CREB

## Abstract

Cancer cells aberrantly express mucins to enhance their survival. Relative chemoresistance of appendiceal pseudomyxoma peritonei (PMP) is attributed to abundant extracellular mucin 2 (MUC2) protein production. We hypothesized that simultaneous MUC2 inhibition and apoptosis induction would be effective against mucinous tumors. *In vitro* studies were conducted using LS174T cells (MUC2-secreting human colorectal cancer cells), PMP explant tissue, and epithelial organoid cultures (colonoids) derived from mucinous appendix cancers. *In vivo* studies were conducted using murine intraperitoneal patient-derived xenograft model of PMP. We found COX-2 over-expression in PMP explant tissue, which is known to activate G-protein coupled EP4/cAMP/PKA/CREB signaling pathway. MUC2 expression was reduced *in vitro* by small molecule inhibitors targeting EP4/PKA/CREB molecules and celecoxib (COX-2 inhibitor), and this was mediated by reduced CREB transcription factor binding to the *MUC2* promoter. While celecoxib (5–40 µM) reduced MUC2 expression *in vitro* in a dose-dependent fashion, only high-dose celecoxib (≥ 20 µM) decreased cell viability and induced apoptosis. Chronic oral administration of celecoxib decreased mucinous tumor growth in our *in vivo* PMP model via a combination of MUC2 inhibition and induction of apoptosis. We provide a preclinical rationale for using drugs that simultaneously inhibit MUC2 production and induce apoptosis to treat patients with PMP.

## INTRODUCTION

Mucinous appendix cancers are a unique histologic subtype in which greater than 50% of the tumor mass is composed of extracellular mucin 2 (MUC2) protein. They frequently lead to the accumulation of large quantities of paucicellular mucinous tumor nodules and mucinous ascites within the abdominal cavity (referred to as pseudomyxoma peritonei [PMP]) [1–4]. MUC2 is a gel-forming glycoprotein that is thought to be secreted by neoplastic goblet-like epithelial cells and produces a mucinous protective environment surrounding the tumor cells [2, 5, 6]. Mucinous histologic subtypes may arise in cancers of the colon, rectum, stomach, ovary, and esophagus and generally have a higher tendency for lymph node and peritoneal metastases. They are less responsive to standard palliative cytotoxic chemotherapeutic drugs and neoadjuvant chemoradiation therapy than their non-mucinous counterparts [3, 7–9]. In general, tumors are known to aberrantly express mucins in order to modulate the tumor microenvironment in favor of cancer cell proliferation, invasion, metastasis, immune evasion and chemoresistance [10]. While the role of MUC2 protein in appendiceal PMP remains unclear, we postulated that the unique mucinous phenotype likely contributes to its distinct tumor biology, clinical behavior and relative chemoresistance.

Mucinous appendix and colorectal cancers demonstrate distinct molecular profiles compared to their non-mucinous counterparts [3, 11, 12]. These unique tumor-associated molecular aberrations may represent therapeutic targets. Published reports, including data from the cancer genome atlas (TCGA), have identified higher rates of *KRAS*, *BRAF*, *PIK3CA*, and *GNAS* mutations in these mucinous subtypes, suggesting distinct molecular pathogenesis [12–14]. These genomic data implicate mitogen-activated protein kinase (MAPK), phosphoinositide 3-kinase (PI3K) and cyclic AMP-dependent protein kinase A (cAMP/PKA) signaling pathways as potential drivers of mucinous tumorigenesis. We hypothesized that inhibiting key molecular drivers of mucinous tumorigenesis would be an effective therapeutic strategy to reduce mucinous tumor growth and perhaps improve the efficacy of standard cytotoxic chemotherapeutic drugs. We have previously published promising preclinical data demonstrating effective reduction of MAPK pathway-mediated MUC2 protein production and mucinous tumor growth *in vitro* and *in vivo* following treatment with MEK (MAP kinase/ERK kinase) inhibitors [15].

In this study, we investigated the impact of inhibiting Prostaglandin E2 (PGE2)/ G-protein coupled E-type prostanoid receptor 4 (EP4)/ cyclic AMP (cAMP)/ protein kinase A (PKA)/ cAMP response element binding protein (CREB) signaling pathway on MUC2 protein production and mucinous tumor growth using *in vitro* models of mucinous appendix cancer/PMP, as well as a unique *in vivo* murine intraperitoneal patient-derived xenograft (PDX) model of PMP, developed in our laboratory [16]. We also provide a mechanistic rationale for using the FDA approved drug celecoxib to inhibit MUC2 protein production and mucinous tumor growth. We studied the cAMP/PKA pathway because *GNAS* mutations (encoding for secretory G-protein-alpha [G_s_-α]) are a common feature of mucinous appendix cancers/PMP and are known to activate cAMP/PKA-mediated CREB (cAMP response element binding protein) transcription factor activity [17–23]. Importantly, the *MUC2* promoter has been shown to harbor a CREB-responsive element (CRE) providing a potential mechanism for cAMP/PKA-mediated modulation of mucin production.(24) We tested the preclinical efficacy of celecoxib in this study because it inhibits cyclooxygenase-2 (COX-2), an enzyme that is overexpressed in mucinous colorectal and appendix cancers [11, 25, 26]. COX-2 inhibition decreases PGE2-mediated EP4 receptor activation and subsequent downstream cAMP/PKA/CREB-mediated transcription [27, 28]. In addition, celecoxib inhibits adenylyl cyclase to decrease cAMP production and therefore PKA activity [25]. Moreover, celecoxib has been shown to induce apoptosis via non-COX-2 targets including 3-phosphoinositide-dependent protein kinase-1 (PDK-1), sarcoplasmic/endoplasmic reticulum calcium ATPase (SERCA) and β-catenin-TCF-LEF complex [29–31]. We hypothesized that mucinous appendix cancers/PMP would demonstrate a particularly favorable treatment response to drugs like celecoxib that simultaneously inhibit MUC2 production and induce apoptosis.

## RESULTS

### COX-2 over-expression in mucinous appendix cancer/PMP

**Figure 1:**
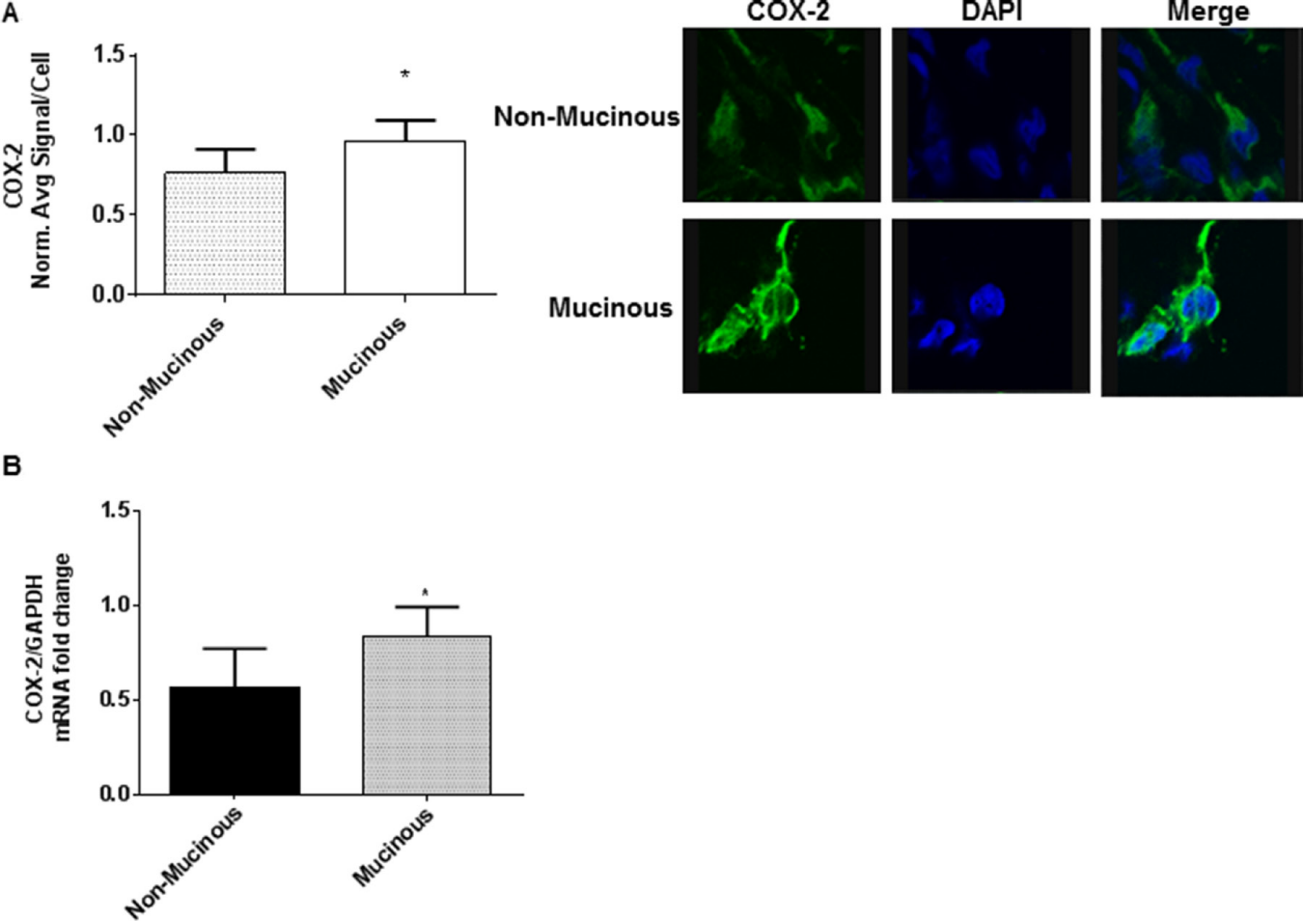
Mucinous appendix cancers/PMP demonstrate COX-2 over-expression Explant tissue from six mucinous appendix neoplasms/PMP patients demonstrated significantly higher levels of COX-2 protein expression (**A**) and mRNA expression (**B**) than non-mucinous appendix cancers. Representative slides from six separate tumor explants samples are shown. Protein expression in explant tissue was measured by IF staining, slides were stained with COX-2 antibody (green IF), SYTOX Orange was used to stain nucleic acid (blue IF), confocal images were randomly taken of 10 different fields (X 63 magnification) and analyzed using Image-pro Premier Software to quantify the average intensity of COX-2 protein expression. Commercially available primers and probe specific for MUC2 and GAPDH cDNA were used for real-time PCR assay; relative amounts of MUC2 mRNA were determined after normalization of mucin transcripts to that of GAPDH. (IF: immunofluorescence).

We found significantly higher COX-2 protein and mRNA expression in mucinous appendix cancer/PMP explant tissue compared to their non-mucinous counterparts. (Figure 1) COX2-mediated PGE2/EP4 receptor activation is known to up-regulate cAMP/PKA/CREB molecular pathway signaling. Importantly, the *MUC2* promoter has been shown to harbor a CREB-responsive element (CRE) providing a potential downstream mechanism for cAMP/PKA/CREB-mediated modulation of mucin production. In addition, previously published data have identified frequent activating *GNAS* mutations and increased PKA activity in mucinous appendix cancers/PMP [17, 18, 20, 22, 32].

### PKA/CREB signaling drives MUC2 production *in vitro*

**Figure 2:**
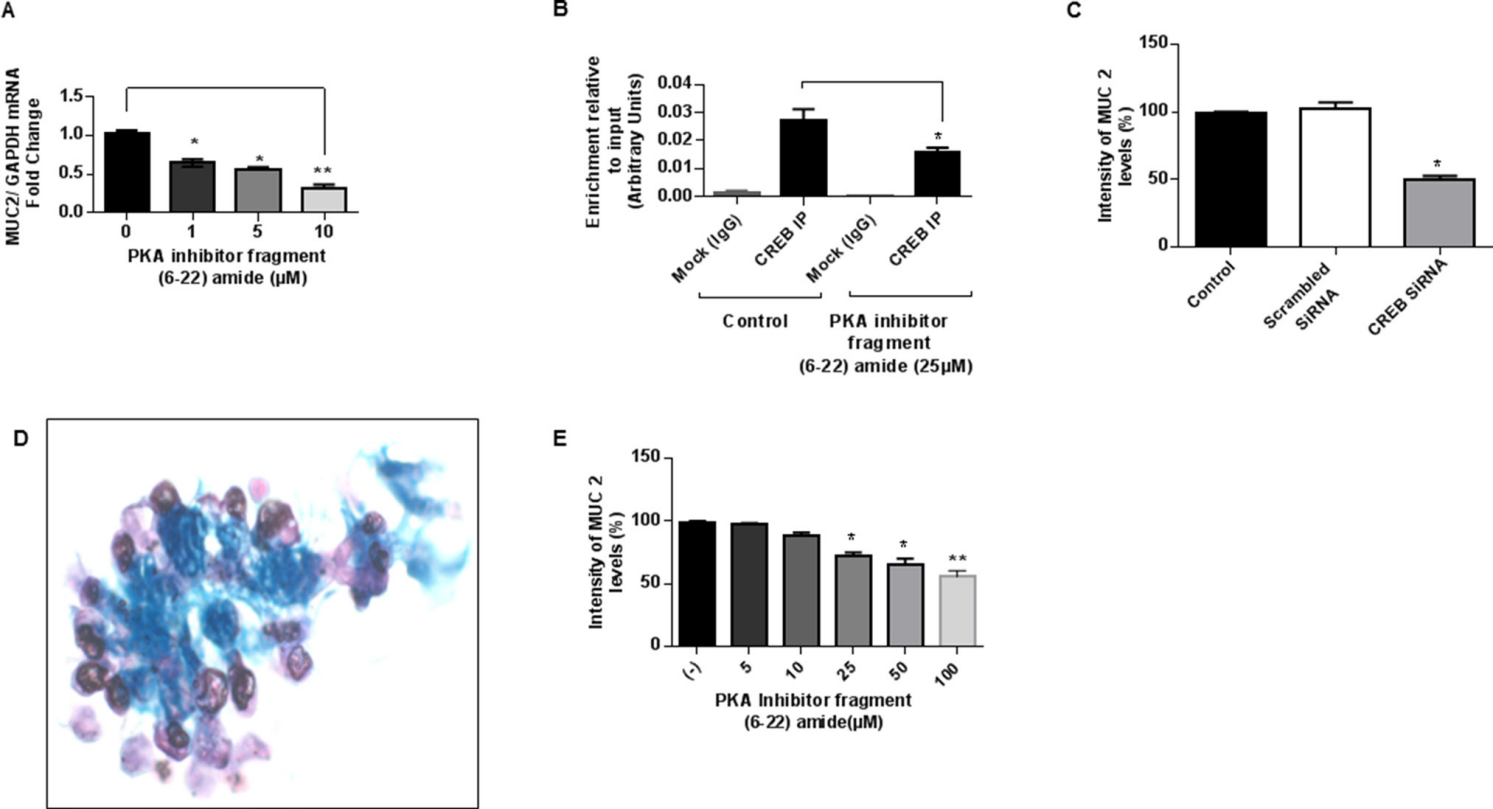
PKA/CREB signaling drives MUC2 production *in vitro* (**A**) PKA inhibitor (fragment 6–22 amide) reduced MUC2 mRNA expression in a dose-dependent manner in LS174T cells at 24 hours; commercially available primers and probe specific for MUC2 and GAPDH cDNA were used for real-time PCR assay; relative amounts of MUC2 mRNA were determined after normalization of mucin transcripts to that of GAPDH. (**B**) PKA inhibitor decreased CREB-transcription factor binding to the *MUC2* promoter at 6 hours as shown by ChIP assay; chromatin solutions were immunoprecipitated (IP) using 4 μg of anti-CREB antibody; for a negative control (mock) rabbit IgG was used. (**C**) MUC2 protein expression was inhibited following exposure of LS174T cells to CREB siRNA (10 mM) compared to scrambled siRNA at 72 h as shown by flow cytometry assay; fixed and permeabilized cells were stained with the MUC2-FITC antibody; intracellular immunostaining was analyzed using Accuri C6 Flow Cytometer. (**D**) Alcian blue staining of *in vitro* colonoid cultures of mucinous appendix cancer grown in matrigel demonstrating abundant extracellular mucin (blue stain). (**E**) PKA inhibitor (fragment 6–22 amide) reduced MUC2 protein expression as shown by flow cytometry assay in colonoid cultures of mucinous appendix cancer at 24 hours. Error bars represent standard error of the mean (SEM) from triplicate experiments. Asterisk represents a statistically significant difference compared with the control group (^*^p < 0.05; ^**^p < 0.01). (PCR: polymerase chain reaction; siRNA: small interfering RNA; ChIP: chromatin immunoprecipitation).

Treatment of LS174T cells with PKA inhibitor (fragment 6–22 amide) decreased MUC2 mRNA expression in a dose-dependent manner, with > 50% inhibition at a dose of 10 µM for 24 hours. (Figure 2A) Moreover, treatment with PKA inhibitor decreased CREB-transcription factor binding to the *MUC2* promoter in LS174T cells, suggesting involvement of PKA/CREB signaling molecules in the regulation of MUC2 production. (Figure 2B) Furthermore, MUC2 protein expression was inhibited following exposure of LS174T cells to CREB siRNA compared to scrambled siRNA, indicating direct involvement of CREB transcription factor in the regulation of *MUC2* promoter activity. (Figure 2C) We sought to confirm some of these findings using *ex vivo* colonoid cultures of mucinous appendix cancer/PMP. (Figure 2D) Treatment of colonoid cultures with PKA inhibitor reduced MUC2 protein expression in a dose-dependent manner. (Figure 2E) These data suggest involvement of the PKA/CREB signaling pathway in modulation of MUC2 production *in vitro* and that this pathway may represent a relevant target for decreasing MUC2 production.

### MUC2 production is modulated by PGE2-mediated cAMP/PKA/CREB pathway activation *in vitro*

**Figure 3:**
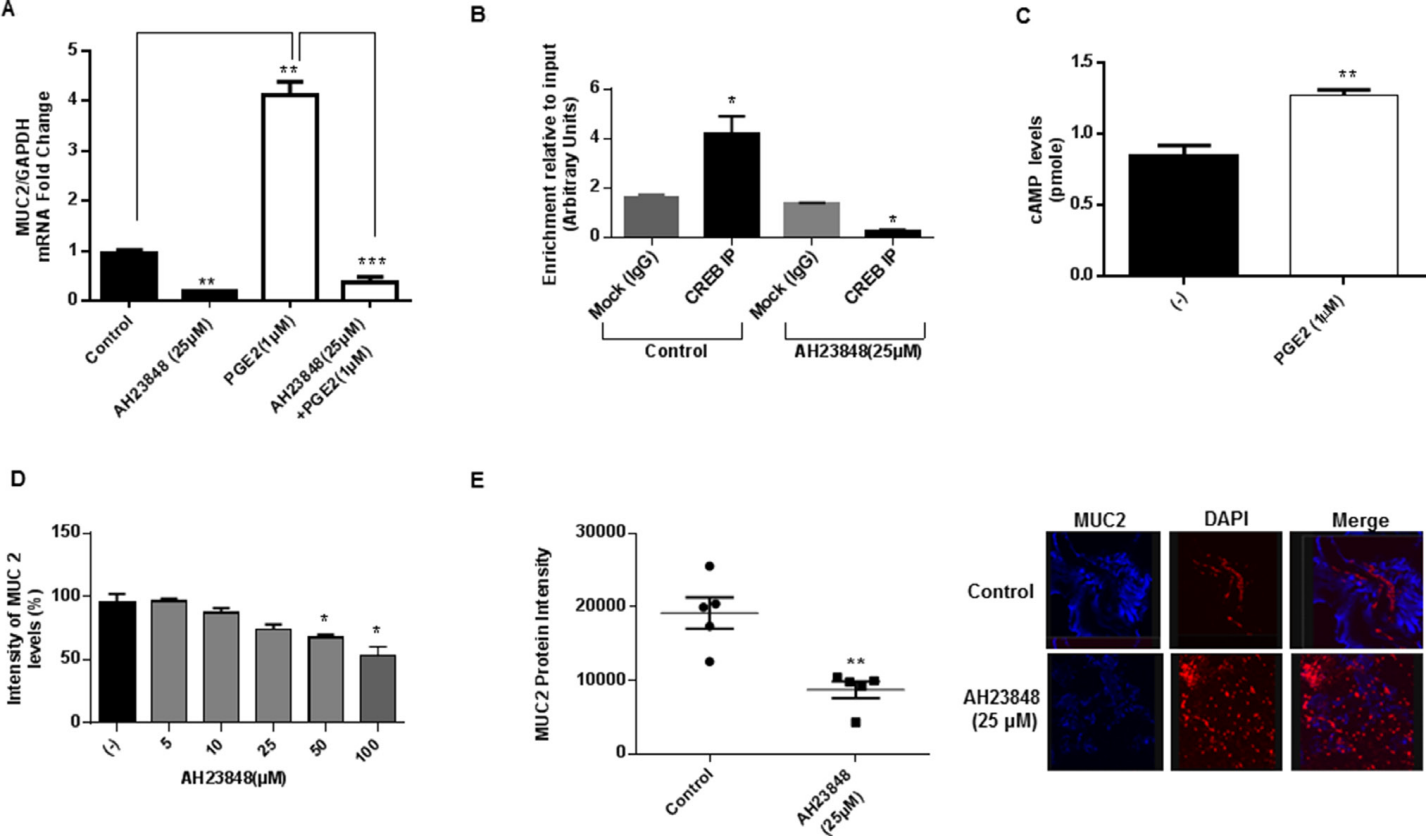
MUC2 production is modulated by PGE2-mediated cAMP/PKA/CREB pathway activation *in vitro* (**A**) G-protein coupled-EP4 receptor inhibitor AH23848 inhibited PGE2-induced MUC2 mRNA expression in LS174T cells at 24 hours; commercially available primers and probe specific for MUC2 and GAPDH cDNA were used for real-time PCR assay; relative amounts of MUC2 mRNA were determined after normalization of mucin transcripts to that of GAPDH. (**B**) AH23848 decreased CREB binding to the *MUC2* promoter in LS174T cells at 6 hours as shown by ChIP assay; chromatin solutions were immunoprecipitated (IP) using 4 μg of anti-CREB antibody; for a negative control (mock) rabbit IgG was used. (**C**) Exposure of colonoid cultures to PGE2 increased cAMP levels at 6 hours, consistent with activation of adenylyl cyclase activity; colonoids were trypsinized and 5000 aggregates per well were plated on to a Matrigel coated 12 well plate. During seeding, PGE2 was added at a final concentration of 1mM. After 6 hours colonoids were collected and examined for cellular cAMP level using a cAMP parameter assay kit. (**D**) AH23848 inhibited MUC2 protein expression in colonoid cultures at 24 hours as shown by flow cytometry assay; fixed and permeabilized cells were stained with the MUC2-FITC antibody; intracellular immunostaining was analyzed using Accuri C6 Flow Cytometer. (**E**) AH23848 inhibited MUC2 protein expression in PMP explant tissue at 24 hours; protein expression in explant tissue was measured by IF staining, slides were stained with MUC2 antibody (blue IF), SYTOX Orange was used to stain nucleic acid (red IF), confocal images were randomly taken of 10 different fields (X 63 magnification) and analyzed using Image-pro Premier Software to quantify the average intensity of MUC2 protein expression. Error bars represent standard error of the mean (SEM) from triplicate experiments. Asterisk represents a statistically significant difference compared with the control group (^*^p < 0.05; ^**^p < 0.01; ^***^p < 0.001). (PCR: polymerase chain reaction; ChIP: chromatin immunoprecipitation; IF: immunofluorescence).

Exposure of LS174T cells to exogenous PGE2 increased MUC2 mRNA expression, while PGE2-induced MUC2 mRNA expression was inhibited by the G-protein coupled-EP4 receptor inhibitor AH23848. (Figure 3A) Moreover AH23848 decreased CREB binding to the *MUC2* promoter in LS174T cells, demonstrating direct involvement of GPCR activity in modulation of MUC2 production. (Figure 3B) We sought to confirm some of these findings using *ex vivo* colonoid cultures of mucinous appendix cancer. Exposure of colonoid cultures to PGE2 increased cAMP levels, consistent with activation of adenylyl cyclase activity and hence downstream cAMP/PKA/CREB signaling. (Figure 3C) Moreover, treatment with AH23848 inhibited MUC2 protein expression in colonoid cultures and PMP explant tissue. (Figure 3D and 3E) These data suggest that COX-2/PGE2 activity may drive MUC2 production via GPCR signaling and CREB-mediated transcriptional activity.

### Celecoxib inhibits MUC2 production *in vitro* via GPCR/cAMP/PKA/CREB pathway

**Figure 4:**
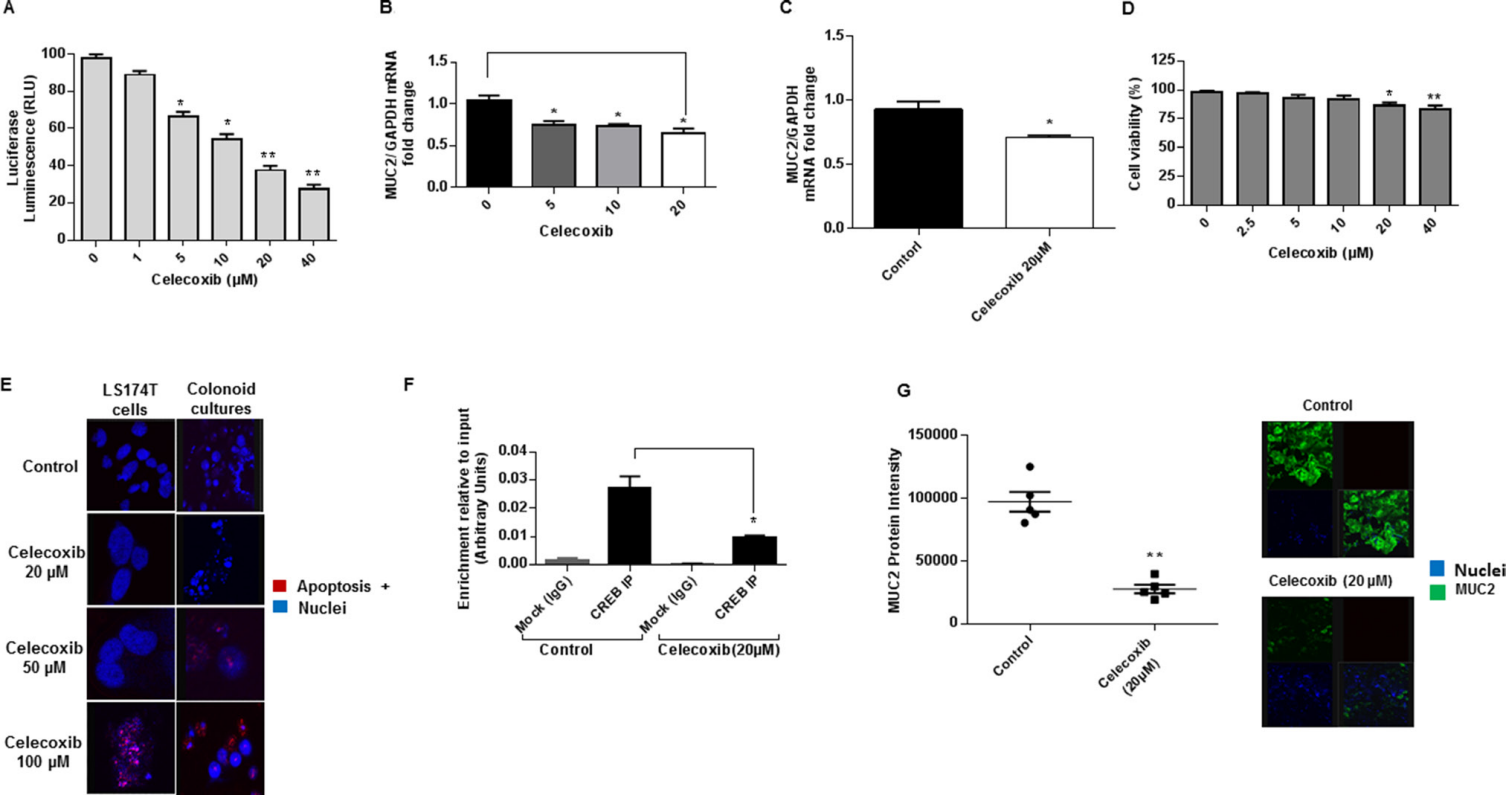
Celecoxib inhibits MUC2 production *in vitro* via GPCR/cAMP/PKA/CREB pathway (**A**) Celecoxib decreased *MUC2* promoter activity in a dose dependent fashion in transient transfected LS174T cells expressing luciferase-labelled *MUC2* promoter construct. Celecoxib decreased MUC2 mRNA expression in LS174T cells (**B**) as well as colonoid cultures (**C**); commercially available primers and probe specific for MUC2 and GAPDH cDNA were used for real-time PCR assay; relative amounts of MUC2 mRNA were determined after normalization of mucin transcripts to that of GAPDH. Treatment with celecoxib decreased cell viability at high doses (≥ 20 µM) (**D**), while *MUC2* promoter activity (A) and mRNA expression (B) were reduced at low and high doses; cell viability was determined by CellTiter 96 aqueous non-radioactive cell proliferation (MTS) assay. (**E**) Celecoxib induced apoptosis in LS174T cells and colonoid cultures by TUNEL assay. (**F**) Celecoxib decreased CREB-transcription factor binding to the *MUC2* promoter at 6 hours in LS174T cells as shown by ChIP assay; chromatin solutions were immunoprecipitated (IP) using 4 μg of anti-CREB antibody; for a negative control (mock) rabbit IgG was used. (**G**) MUC2 protein expression was reduced in mucinous tumor explant tissues following *ex vivo* treatment by celecoxib; protein expression in explant tissue was measured by immunofluorescence (IF) staining, slides were stained with MUC2 antibody (green IF), SYTOX Orange was used to stain nucleic acid (blue IF), confocal images were randomly taken of 10 different fields (X 63 magnification) and analyzed using Image-pro Premier Software to quantify the average intensity of MUC2 protein expression. Error bars represent standard error of the mean (SEM) from triplicate experiments. Asterisk represents a statistically significant difference compared with the control group (^*^p < 0.05; ^**^p < 0.01). (PCR: polymerase chain reaction; ChIP: chromatin immunoprecipitation).

Using transiently transfected LS174T cells expressing *MUC2* promoter-luciferase reporter construct, we found that celecoxib decreased *MUC2* promoter activity in a dose dependent manner. (Figure 4A) In addition, celecoxib decreased MUC2 mRNA expression in LS174T cells and colonoid cultures. (Figures 4B and 4C) We performed cell viability assay to confirm that the MUC2 inhibitory effect of celecoxib was independent of potential cytotoxicity. We demonstrated significant reduction in MUC2 mRNA expression at low and high doses of celecoxib, while cell viability was effected at high doses (≥ 20 µM). (Figures 4B and 4D) We used TUNEL assay in LS174T cells and colonoid cultures to confirm that celecoxib-induced apoptosis occurred at high doses only. (Figure 4E) These data would suggest that celecoxib has a dual inhibitory effect on MUC2 production and cell survival. Since celecoxib is known to decrease COX-2 activity and hence PGE2 synthesis, we investigated whether its MUC2 inhibitory effects occurred via the cAMP/PKA/CREB pathway. This was confirmed using ChIP assay in which we demonstrated a reduction in CREB-transcription factor binding to the *MUC2* promoter of LS174T cells following treatment with celecoxib. (Figure 4F) We sought to confirm the efficacy of celecoxib in PMP explant tissue; *ex vivo* treatment of PMP explant tissue with celecoxib reduced MUC2 protein expression. (Figures 4G) These data suggest that celecoxib inhibits MUC2 production *in vitro* via GPCR/cAMP/PKA/CREB pathway regardless of cytotoxicity and provides a preclinical rational for the use of celecoxib to control the growth of mucinous tumors like PMP given its MUC2 suppressive and cytotoxic properties.

### Celecoxib inhibits mucinous tumor growth via cAMP/PKA signaling pathway *in vivo*

**Figure 5:**
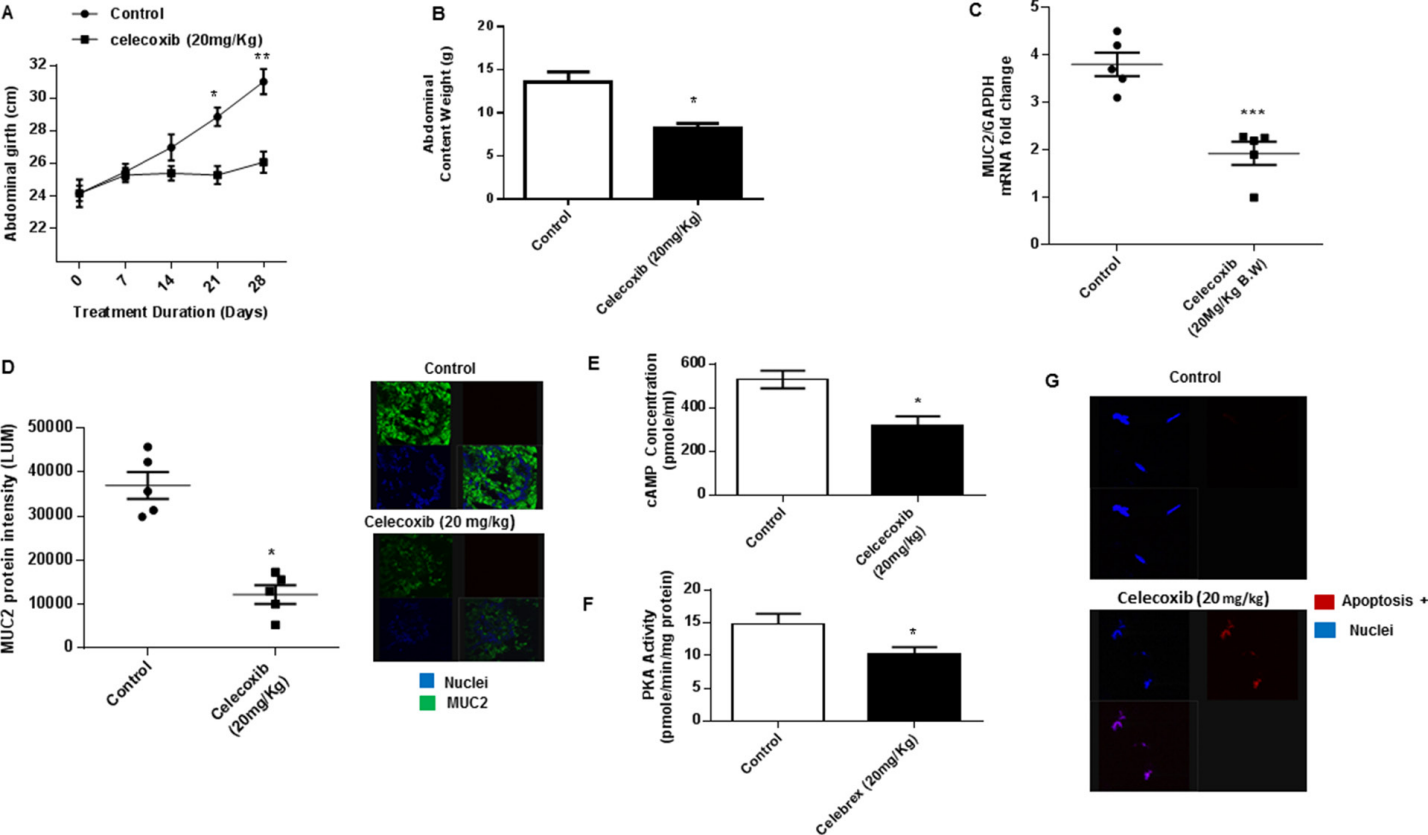
Celecoxib inhibits mucinous tumor growth via cAMP/PKA signaling pathway *in vivo* (**A**) Chronic oral gavage with celecoxib (20 mg/kg, every other day) for 28 days reduced mucinous tumor growth in our *in vivo* PDX model of PMP (compared to control animals treated with oral PBS gavage; 6 animals per group); serial measurements were taken of abdominal girth (mm) over the duration of treatment. (**B**) Intraperitoneal tumor burden (abdominal contents measured in grams) at the time of sacrifice was significantly smaller in the celecoxib-treated animals compared to control animals. Analysis of the excised tumor tissue following sacrifice demonstrated significant reduction of MUC2 mRNA expression (**C**) and protein expression (**D**); commercially available primers and probe specific for MUC2 and GAPDH cDNA were used for real-time PCR assay; relative amounts of MUC2 mRNA were determined after normalization of mucin transcripts to that of GAPDH; protein expression in tumor tissue was measured by IF staining, slides were stained with MUC2 antibody (green IF), SYTOX Orange was used to stain nucleic acid (blue IF), confocal images were randomly taken of 10 different fields (X 63 magnification) and analyzed using Image-pro Premier Software to quantify the average intensity of MUC2 protein expression. Analysis of the excised tumor tissue following sacrifice demonstrated significant reduction of cAMP concentration (**E**) and PKA activity (**F**) when compared to control mice. (**G**) Celecoxib significantly induced apoptosis in treated tumor tissue compared to controls; TUNEL assay was used to identify apoptotic cells. (Figure 5G) Error bars represent standard error of the mean (SEM) from triplicate experiments. Asterisk represents a statistically significant difference compared with the control group (^*^p < 0.05; ^**^p < 0.01; ^***^p < 0.001). (PMP: pseudomyxoma peritonei; PCR: polymerase chain reaction; IF: immunofluorescence).

Using the *in vivo* PDX model we found that chronic oral treatment with celecoxib significantly reduced mucinous tumor growth, as demonstrated by serial measurements of abdominal girth over the duration of treatment, compared to control animals. (Figure 5A) Similarly, intraperitoneal tumor burden at the time of sacrifice (measured by abdominal content weight in grams) was significantly less in the celecoxib-treated animals compared to control animals. (Figure 5B) Analysis of the excised tumor tissue following sacrifice demonstrated significant reduction of MUC2 mRNA and protein expression, suggesting that celecoxib effectively inhibited mucin production *in vivo*. (Figures 5C and 5D) Moreover, cAMP concentration and PKA activity were decreased in celecoxib-treated tumor tissue compared to untreated controls, suggesting that celecoxib inhibited mucinous tumor growth through a reduction in cAMP/PKA signaling pathway activity. (Figures 5E and 5F) At the same time celecoxib significantly induced apoptosis in treated tumor tissue, confirming our *in vitro* findings of dual inhibitory effects of celecoxib on MUC2 production and cell viability. (Figure 5G)

## DISCUSSION

Mucinous appendix cancers/PMP are characterized by abundant extracellular MUC2 protein and demonstrate distinct molecular profiles compared to their non-mucinous counterparts [2, 3, 5, 6, 12]. While the role of MUC2 protein in these tumors remains unclear, the mucinous phenotype is thought to provide a protective environment for cancer cells to thrive [10]. The extracellular mucinous component plays an especially significant role in the clinical course of patients with appendiceal PMP, since massive quantities of intraabdominal mucinous tumor accumulation, characteristic of PMP, causes obstructive organ dysfunction, inanition and death from mass-effect. We therefore postulated that these mucinous cancers would benefit from a therapeutic strategy that inhibited mucin production, and that these tumors would perhaps be especially vulnerable to drugs that simultaneously inhibited mucin production and induced apoptosis. In this study, we targeted the PGE2/EP4/cAMP/PKA/CREB signaling pathway since it is up-regulated in mucinous appendix cancers/PMP as a result of activating *GNAS* gene mutations and COX-2 over-expression [17, 18, 20–23, 26]. We used *in vitro* and *in vivo* models of mucinous appendix cancer/PMP to conduct our experiments. We demonstrated that cAMP/PKA signaling pathway modulated MUC2 production and would therefore be a viable target for therapy. Moreover, we provided a preclinical rationale for the use of celecoxib to suppress mucinous tumor growth since it simultaneously inhibited cAMP/PKA/CREB-mediated MUC2 production and induced apoptosis.

*GNAS* gene mutations are frequently found in a variety of malignancies, especially secretory tumors arising in exocrine and endocrine organs like mucinous colorectal and appendix cancers, suggesting oncogenic and secretory roles for this gene [17, 18, 20, 22, 23, 32]. Published data demonstrate frequent activating *GNAS* gene mutations in mucinous colorectal and appendix cancers. *GNAS* gene encodes the alpha subunit of the heterotrimeric secretory G-protein (G_s_-α) associated with G-protein coupled receptors (GPCRs), which then activates adenylyl cyclase activity and subsequent cAMP/PKA/CREB signaling [18, 19]. We were therefore interested in studying the role of this molecular signaling pathway in the regulation of MUC2 production and as a potential therapeutic target in mucinous colorectal and appendix cancers, especially since the *MUC2* promoter has been shown to harbor a CREB-responsive element (CRE) providing a potential mechanism for cAMP/PKA-mediated modulation of mucin production [24]. Nishikawa and colleagues previously demonstrated cAMP-mediated elevation of MUC2 and MUC5AC expression in stably transfected HT29 colorectal cancer cell line expressing *GNAS*, thereby supporting the role for *GNAS* expression and cAMP/PKA signaling pathway in the regulation of mucin production [21]. Our data demonstrate involvement of the cAMP/PKA/CREB signaling pathway in the modulation of MUC2 expression using *in vitro* and *in vivo* models of mucinous appendix cancer/PMP. It is important to note however that while protein kinases have become very attractive drug targets, clinically applicable inhibitors are not yet available [33].

COX-2 expression and hence PGE2 levels are frequently elevated in colorectal and appendix cancers, especially the mucinous subtypes [11, 26]. The inflammatory mediator PGE2 has been shown to play a role in cancer cell proliferation, apoptosis, invasion and metastasis [28]. PGE2 has also been shown to induce MUC2 production in tracheobronchial and biliary epithelial cell cultures [34, 35]. PGE2 binds to the extracellular domain of G-protein coupled E-type prostanoid receptors EP2 and EP4 and activates downstream cAMP/PKA signaling [18, 19, 36]. In this study we used the FDA approved drug celecoxib to study the role of COX-2 and cAMP/PKA signaling in modulating MUC2 production since it simultaneously inhibits COX-2 and adenylyl cyclase activities [25, 29–31]. Our data demonstrated involvement of the COX-2/PGE2/EP4/cAMP/PKA/CREB signaling pathway in the modulation of MUC2 expression in both *in vitro* and *in vivo* models of mucinous appendix cancer/PMP. Moreover, we provided promising preclinical data to support the use of celecoxib to decrease mucinous tumor growth *in vivo*, as a result of its ability to simultaneously suppress MUC2 production and promote apoptosis. Celecoxib has been extensively evaluated for the prevention and treatment of a variety of cancers, including colorectal cancer, in light of its anti-tumor effects through COX-2 and more importantly non-COX-2 target activities [37–39].

An important aspect of this study is the use of adult stem cell based *ex vivo* colonoid cultures and tumor explant tissue derived from patients with mucinous appendix cancers/PMP to confirm the results seen in the mucin secreting LS174T cells. Such *ex vivo* cultures and tissue explants are more representative of the cytohistopathologic and genetic diversity of *in situ* human tumors [40]. Furthermore, the *in vivo* PDX model of PMP used in this study provides a unique opportunity for drug testing on individual patient tumors that may have distinct phenotypic and genetic characteristics [16]. We have previously demonstrated that our PDX model of PMP mimics the histopathology and clinical course of the disease in patients and is therefore an ideal model for such studies [15, 41, 42].

Traditionally, chemotherapeutic and biologic agents are used to target neoplastic epithelial cell in order to control cancer growth. However, patients with mucinous appendix cancers/PMP tend to be relatively chemoresistant compared to their non-mucinous counterparts [3, 9]. This chemoresistance has been partially attributed to the protective barrier formed by the extracellular MUC2 protein surrounding the cancer cells. This is especially true in the case of appendiceal PMP in which massive amounts of extracellular mucin accumulates within the abdominal cavity. In addition, the clinical course of these paucicellular tumors is frequently determined by the mucinous component, which causes morbidity and mortality from mass-effect rather than cellular invasion. MUC2 secretion by neoplastic goblet-like cells in PMP maybe an adaptive mechanism that supports cell survival, allows immune evasion and provides a growth-supportive microenvironment. We postulate that a treatment strategy that simultaneously inhibits mucin production and induces apoptosis may overcome some of the unique clinical challenges posed by these mucinous tumors. For example, we may be able to reduce the compressive symptoms related to mucinous tumor growth and perhaps improve the efficacy of systemic or regionally delivered chemo-immunotherapies by exposing the neoplastic epithelial cells embedded within the mucinous deposits. Future studies will determine whether this treatment strategy improves chemosensitivity to standard chemotherapeutic agents.

In conclusion, our results showed that MUC2 production in mucinous appendix cancer/PMP was partially regulated through the COX-2/PGE2/GPCR/cAMP/PKA/CREB signaling pathway. MUC2 production could be reduced in *ex vivo* colonoid cultures and *in vivo* PDX models derived from PMP tissue. Treatment with celecoxib effectively reduced mucinous tumor growth inhibition *in vivo*, supporting our hypothesis that mucinous tumors may be especially vulnerable to a therapeutic strategy that simultaneously suppresses MUC2 suppression and induces apoptosis.

## MATERIALS AND METHODS

### Reagents

DMEM (Dulbecco’s Modified Eagle’s Medium) was obtained from Invitrogen (Carlsbad, CA). Fetal bovine serum (FBS) was obtained from Hyclone laboratories (Logan, UT). Cell-culture plates were purchased from Costar (Cambridge, MA). Celecoxib, G-protein coupled-EP4 receptor inhibitor AH23848, protein kinase inhibitor (fragment 6–22 amide), and prostaglandin E2 (PGE2) were obtained from Cayman chemical (Ann Arbor, MI). siRNA for CREB and RNeasy Mini Kit were obtained from Qiagen (Valencia, CA). CREB antibody for ChIP (Chromatin Immunoprecipitation) assay was obtained from Millipore (MA). CellTiter 96 Aqueous Assay was obtained from Promega (Madison, WI). Assay for measuring cAMP concentration was obtained from R&D Systems (Minneapolis, MN) and PKA activity assay were obtained from Arbor Assays Headquarters (Ann Arbor, MI). Basement membrane matrix (Matrigel) was obtained from (Corning, Massachusetts). Female athymic nude mice were obtained from Taconic (Tarrytown, NY). Reverse transriptase-polymerase chain reaction (RT-PCR) kits, including primers and probe for MUC2 and glyceraldehyde 3-phosphate dehydrogenase (GAPDH), were obtained from Applied Biosystems (ABI, Foster City, CA). The enhanced chemiluminescence reagents (ECL) kit and Pierce BCA protein assay kit were obtained from ThermoScientific (Rockford, IL). CREB antibody for western blot and immunofluorescence assay was obtained from BD Biosciences (Rockford, IL). MUC2 antibody for immunofluorescence assay and anti-rabbit and anti-mouse horseradish peroxidase (HRP)-conjugated secondary antibodies were purchased from Santa Cruz Biotechnology (Santa Cruz, CA). Tissue Path Disposable Base Molds, Tissue-Tek O.C.T compound Superfrost Plus microscope slides were obtained from Fisher Scientific (Pittsburgh, PA). COX-2 antibody for immunofluorescence assay was obtained from Abcam, Anti-rabbit Alexa 647 and Alexa 488 were obtained from Cell Signaling Technology (Danvers, MA). SYTOX Orange for nucleic acid labeling was obtained from Life Technologies (Grand Island, NY). MUC2-FITC antibody for flow cytometric assay was obtained from MyBioSource (San Diego, CA).

### Cell culture and treatment

LS174T cells were obtained from American Type Culture Collection (Manassas, VA). These cells were originally derived from a human mucinous colorectal cancer and demonstrate “characteristics of goblet-cells” by secreting relatively high levels of MUC2 protein. It is a well-established cell line for studying the regulation of MUC2 expression. LS174T cells were grown in cell-culture plates in DMEM (supplemented with 4.5 g/L glucose, 10% fetal bovine serum, 2 mM L-glutamine, 20 mM HEPES, 100 IU/ml penicillin and 100 µg/ml streptomycin) at 37°C and 5% CO_2_. Pre-confluent (60–70% confluent) LS174T cells were exposed to the varying concentrations of drugs, including celecoxib (0–40 µM), AH23848 (0–100 µM), fragment 6–22 amide (0–100 µM), PGE2 (1 µM) for varying time-periods. For the controls, LS174T cells were incubated with medium alone for the same amount of time. Viability of cells (> 95%) was confirmed using trypan blue staining.

### Generation of *ex vivo* epithelial organoid cultures (i.e. colonoids)

Fresh primary mucinous appendix cancer tissue was used to develop *ex vivo* epithelial organoid cultures (colonoids) [40]. Mucosa was stripped of the underlying muscle layer and tumor tissue fragments were washed and incubated in chelation solution supplemented with EDTA (2 mM final concentration). Basal culture medium (advanced Dulbecco’s modified Eagle medium/F12 supplemented with penicillin/streptomycin, 10 mM HEPES, and Glutamax) was added, and the crypts were washed twice with basal culture medium and suspended in Basement Membrane Matrix (BMM). The BMM was polymerized during incubation at 37°C, 5% CO2 incubator for 30 minutes. The BMM was overlaid with human intestinal stem cell medium.

### PMP explant tissue processing and treatment

Fresh PMP tumor tissue was delivered to the laboratory on ice within 30 minutes of resection for processing, under an approved Institutional Review Board protocol at the University of Pittsburgh (UPCI IRB# 02–077). Tissue was dissected with a scalpel into uniform blocks of 2 mm^3^ dimensions and placed in tissue culture plates containing the same medium used for LS174T cell culture. Explant tissue from three to six patients was exposed to the indicated concentrations of drugs. For the controls, explant tissue were incubated with medium alone for the same time period.

### Intraperitoneal patient-derived xenograft (PDX) model

Development of our intraperitoneal murine xenograft model has been published [16] Fresh PMP tumor was processed and implanted in the peritoneal cavity of nude mice. The resulting model has been successfully passaged to subsequent generations in nude mice with 100% reliability and retains the clinical and pathologic characteristics of the original human tumor. Mucinous tumor growth becomes clinically at 2 weeks with progressive increase in abdominal girth and body weight over the following weeks. Animals were randomized at day 7, following tumor inoculation, to different treatment groups (6 animals per group) and weekly measurements of gross body weight (grams) and abdominal girth (millimeters) were recorded. Following completion of experiments, animals were sacrificed and abdominal contents (abdominal organs + mucinous tumor deposits) were harvested en-bloc and weighed.

### LS174T cells expressing *MUC2* promoter-luciferase reporter construct

MUC2-luciferase reporter plasmid was obtained from SwitchGear Genomics (Carlsbad, CA) and transiently transfected into LS174T cells seeded at a concentration of 6 × 10^5^ cells per well in 6-well plates using Lipofectamine 2000 (Life Technologies, Grand Island, NY), following the manufacturer’s protocol. After 24 hours of exposure to the transfection mixture, the cells were incubated in medium containing 10% FBS and celecoxib for an additional 18 hours and then harvested for measurement of luciferase activity by Promega Luciferase assay system (Madison, WI).

### Reverse transcription (RT) and real-time polymerase chain reaction (real-time PCR) analysis

Total RNA was isolated from harvested LS174T cells, colonoids or human PMP tissue using RNeasy Mini Kit and quantified using Nanodrop ND-1000 spectrophotometer (Wilmington, DE). Each sample was reverse transcribed into cDNA in a Peltier Thermal Cycler (PTC-220 DNA Engine Dyad, MJ Research; Waltham, MA) using random hexamers and the GeneAmp RNA PCR Core Kit (ABI). Real-time PCR was then carried out in an ABI Prism SDS 7000 Cycler System (ABI), using commercially available primers and probe obtained from ABI, specific for MUC2 and GAPDH cDNA, for 40 cycles at 95°C for 15 seconds. Relative amounts of MUC2 mRNA were determined after normalization of mucin transcripts to that of GAPDH, using software supplied by the manufacturer (ABI).

### Immunofluorescence assay

Cells or tissue were placed in Tissue Path Disposable Base Molds and snap frozen in Tissue-Tek O.C.T compound. Using a cryostat microtome, 5 micron frozen sections of tumor tissue were mounted on Superfrost Plus microscope slides and maintained at –20°C. The slides were incubated in 4% paraformaldehyde for 15 minutes, washed, and blocked for 60 minutes at room temperature. The slides were then stained for 3 hours at room temperature with MUC2 or COX-2 antibody. The slides were washed 3 times with 1X PBS and incubated with anti-rabbit Alexa 647 or Alexa 488 and SYTOX Orange for nucleic acid staining for 30 minutes at room temperature. The slides were washed 3 times with 1X PBS and once with high-salt PBS. Cover slips were mounted on the sections using ProLong Gold antifade solution from Invitrogen (Life Technologies, Grand Island, NY). *In situ* apoptosis in explant tissue, colonoid cultures and LS174T cells was detected by TUNEL (terminal deoxynucleotidyl transferase dUTP nick end labeling) using *in situ* BrdU-Red DNA fragmentation assay kit (ab66110, Abcam, Cambridge, MA) according to the manufacturer’s protocol. Confocal images were randomly taken of 10 different fields (X 63 magnification) using a LEICA confocal TCS SL DMRE microscope. Images of each slide were then analyzed using Image-pro Premier Software to quantify the average intensity of MUC2 or COX-2 expression.

### Flow cytometric analysis

Intracellular immunostaining analyses were performed using an Accuri C6 Flow Cytometer. LS174T cells were stained with the MUC2-FITC antibody. Before staining, cells were fixed for 15 minutes using fixing reagent (Leucoperm, Bio-Rad, CA), following which intracellular staining was performed by placing cells in permeabilization reagent (Bio-Rad, CA) along with MUC2-FITC antibody. Cells were stained for 30 minutes at room temperature, followed by washing in PBS supplemented with 0.5% BSA and 0.1% NaN3, then fixed and stored in 1% paraformaldehyde until analysis.

### Chromatin immunoprecipitation (ChIP) assay

ChIP analysis was performed following a protocol provided by Qiagen under modified conditions. LS174T cells were cross linked by adding 1.0% formaldehyde buffer containing 100mM sodium chloride, 1 mM EDTA-Na (pH 8.0), 0.5 mM EGTA-Na, Tris-HCl (pH 8.0) directly to culture medium for 10 minutes at 37 °C. The medium was aspirated, the cells were washed using ice-cold PBS containing 10 mM DTT and protease inhibitors. The cells were then lysed with lysis buffer and incubated for 10 minutes on ice. The cell lysates were sonicated to shear DNA and the samples were diluted to 10-fold in ChIP dilution buffer (0.01% SDS, 1.1% Triton X-100, 1.2 mM EDTA, 16.7 mM Tris, pH 8.1, 167 mM NaCl). To reduce nonspecific background, cell pellet suspension was pre-cleared with 50 μl of Protein-A beads for 1 hour at 4°C with agitation. Chromatin solutions were precipitated overnight at 4°C using 4 μg of anti-CREB antibody with rotation. For a negative control, rabbit IgG was used. 50 μl of Protein-A-agarose slurry was added for 2 hours at 4°C with rotation to collect the antibody-histone complex and washed extensively following the manufacturer’s protocol. Input and immunoprecipitated chromatin were incubated at 65°C overnight to reverse cross-linking. After proteinase K digestion for 1 hour, DNA was extracted using a Qiagen spin column kit. Precipitated DNA was analyzed by PCR of 30 cycles.

### Transfection of LS174T with CREB siRNA

LS174T cells (60–70% Confluency) cultured in 35-mm dishes were transfected with Turbofect (ThermoScientific Inc., Rockford, IL) with CREB siRNA (100 nM) or scrambled siRNA (100 nM) (Qiagen, Valencia, CA) in serum-free DMEM medium according to the manufacturer’s recommendation. Six hours post-transfection, 1 ml of fresh complete DMEM was added, and cells were cultured for an additional 48 hours. CREB siRNA or scrambled siRNA transfected cells were exposed to PGE2 for 8 hours. Subsequently MUC2 protein was analyzed by flow cytometry.

### PKA activity and cAMP assay

The measurement of PKA activity was performed using the PKA Colorimetric Activity Kit (Arbor Assays Headquarters, Ann Arbor, MI) following manufacturer’s instructions. Briefly, tumor tissue from the PDX experiment was sonicated in activated cell lysis buffer provided by the kit, containing 1 mmol/L PMSF, 1 μL/mL protease and phosphatase inhibitor cocktail (Sigma) and incubated for 30 minutes on ice. The lysates were transferred to 1.5 mL reaction tubes and centrifuged at 14,000 g for 15 min at 4°C. The cytosolic fraction was obtained and used as sources for the PKA enzymes. PKA phosphorylated the immobilized PKA substrate on the 96-well microtiter plate in the presence of ATP. The specific antibody of phospho-PKA substrate bound to the immobilized phosphorylated substrate and was detected by peroxidase-conjugated anti-rabbit IgG. After incubation, the intensity of the developed color is proportional to the PKA activity. Cellular cAMP levels in colonoids or xenograft tumor samples was measured using a Mouse/Rat cAMP Parameter Assay Kit (R&D Systems Inc., Minneapolis, MN).

### Cell proliferation assay

Cells were counted and seeded in 96- well microplates overnight. Cells were treated with or without celecoxib for 24 hours. Following treatment, cell viability was determined by CellTiter 96 aqueous non-radioactive cell proliferation (MTS) assay according to the manufacturer’s instructions (Promega, Madison, WI). Cells were treated with a combined solution of a tetrazolium compound MTS [3-(4,5-dimethylthiazol-2-yl)-5-(3-carboxymethoxyphenyl)-2-(4-sulfophenyl)-2H-tetrazolium, inner salt] and an electron coupling reagent PMS (phenazine methosulfate) for additional 2 hours at 37°C. The absorbance of the formazan product at 490 nm was measured directly with an enzyme-linked immunosorbent assay plate reader.

### Tdt-mediated dUTP nick end labeling (TUNEL) assay

Control and treated xenograft tumor tissues were placed in frozen tissue matrix and serial sections 5 microns thick were placed onto slides and stained for TUNEL assay. Sections were deparaffinized and apoptotic cells were detected using the *in situ* BrdU-Red DNA fragmentation (TUNEL) assay kit (Abcam) and counterstained with DAPI.

### Statistical analysis

SPSS (version 21; SPSS Inc., Chicago, IL, USA) was used for performance of statistical analysis. Experimental means were reported ± standard error of the mean (SEM). Data were analyzed with paired Student’s *t*-test. Values were considered significantly different if *p* < 0.05.

## Abbreviations

MUC2: mucin 2
COX-2: cyclooxygenase-2
CREB: cAMP response element binding protein
PKA: protein kinase A
PMP: pseudomyxoma peritonei
EP4: E-type prostanoid receptor
PGE2: prostaglandin E2
MAPK: mitogen-activated protein kinase
PI3K: phosphoinositide 3-kinase
TCGA: the cancer genome atlas
ATCC: American tissue type culture collection
PDX: patient derived xenograft
SERCA: sarcoplasmic/endoplasmic reticulum calcium ATPase
DMEM: Dulbecco’s Modified Eagle’s Medium
